# Recurrent Amaurosis Fugax Secondary to Tolosa-Hunt Syndrome: A Case Report and Review of Phenotypes and Pathology

**DOI:** 10.7759/cureus.15281

**Published:** 2021-05-27

**Authors:** Hassan Kesserwani, Marnix Heersink

**Affiliations:** 1 Neurology, Flowers Medical Group, Dothan, USA; 2 Ophthalmology, Eye Center South, Dothan, USA

**Keywords:** aura, cavernous carotid, headache disorders, scintillating scotoma, igg 4 disease

## Abstract

Tolosa-Hunt syndrome (THS) is a fascinating condition that is ipso facto a cavernous sinus syndome. As such it is associated with inflammation of the cavernous sinus walls and contents with spread to contiguous structures such as the orbital apex and superior orbital fissure. Therefore it does not come as a surprise that there is overlap with the condition of orbital pseudotumor. Furthermore, the typical presentation of THS involves variable affliction of the contents of the cavernous sinus with ocular and facial pain, ophthalmoplegia, facial numbness and Horner syndrome. To our knowledge, we present one of the only reported cases of recurrent amaurosis fugax and transient visual obscurations secondary to THS. Despite being an odd-ball presentation, these manifestations make intuitive sense as we demonstrate luminal narrowing of the right carotid siphon in the setting of cavernous wall enhancement, peri-arteritis of the carotid siphon being well-documented pathologically in the literature. The basis for the transient visual obscurations is more speculative but worthy of further study.

## Introduction

Tolosa-Hunt syndrome (THS) is a cavernous sinus syndrome with or without involvement of the orbital apex or superior orbital fissure. THS is pathologically characterized by granulomatous inflammation of the cavernous sinus walls and immunoglobulin IgG4-related inflammation [1,2]. A malignant invasion of the walls of the cavernous sinus and a carotid siphon aneurysm must be excluded. A Wasserman test to rule out meningovascular syphilis is of historical interest. Nevertheless, the sine qua none of THS is inflammation of the walls of the cavernous sinus by radiological imaging or tissue diagnosis (non-caseating granuloma). Being of an inflammatory nature, steroid-responsive with resolution of symptoms is a necessary but not an absolute prerequisite as lymphomas can respond to steroids [3].

The lateral wall of the cavernous sinus is known as the membranous layer and consists of two separable layers, the dura matter and a dural layer from the sleeves of cranial nerves III, IV and VI. The medial wall, of trapezoidal shape, is a single-layered dural lining and has a sphenoidal and sellar component. The cavernous sinus is filled with venous blood from at least four sources including the superior ophthalmic veins. Its contents include cranial nerves III, IV, V (ophthalmic and mandibular divisions) and VI, the carotid siphon and surrounding oculosympathetics. Therefore a lesion of the cavenous sinus can lead to orbital congestion (proptosis) and chemosis (eyelid swelling and conjunctival injection), ophthalmoplegia with diplopia, eye pain, facial numbness and Horner syndrome. In fact, a sixth nerve palsy and Horner syndrome is localizing of a lesion of the cavernous sinus as the sixth cranial nerve is immediately adjacent to the peri-carotid sympathetics. Furthermore, the absence of mydriasis with a complete oculomotor nerve palsy may implicate the pericarotid sympathetics [4].

The International Headache Society (IHS) criteria for THS are 1) unilateral orbital or peri-orbital headache preceding an ophthalmoplegia involving cranial nerves III, IV or VI, within two weeks; 2) exclusion of other pathology such as sarcoid, malignancy, vasculitis or diabetic-induced fungal infection; 3) evidence of granulomatous inflammation on the same side of the pain by tissue diagnosis or magnetic resonance imaging (MRI) with enhancement of the cavernous sinus, superior orbital fissure and/or orbital apex; 4) steroid-responsiveness is the rule but is not universal [1].

In his original description of six cases, Hunt emphasized the granulomatous lesions of the cavenous sinus walls, the cranial nerve adventitia and peri-carotid adventitia (based upon pathology of surgical specimens), the recurrent and episodic nature of the condition and the importance of excluding a carotid siphon aneurysm by angiography. Adhesion of the cranial nerves to the carotid artery was also reported with invasion by plasma cells and lymphocytes with a scarcity of angiogenesis (non-caseating granuloma) and meningo-vascular syphilis had to be excluded. He also emphasized its phenotypic similarity to ophthalmoplegic migraine, the latter being transitory [5].

Tolosa stressed the need to exclude a carotid siphon aneurysm as the latter may also manifest with trigeminal ophthalmic pain and oculomotor nerve palsy, with variable involvement of cranial nerves IV and VI. In his patient, surgical exploration of the cavernous sinus revealed granulomatous tissue engulfing the carotid siphon with luminal narrowing. After the patient died, pathological sections revealed granulomatous thickening of the adventitia of the carotid siphon [6]. In a study of four cases of the cavernous sinus syndrome responsive to steroids, parasellar tumors were ultimately diagnosed further emphasizing that THS is a diagnosis of exclusion [7]. With advances in imaging technology and increased recognition of THS, bilateral involvement of the cavernous sinus has been increasingly recognized [8,9]. 

## Case presentation

A 75-year-old man presented with the insidious onset of a six-month unremitting constant bi-frontal pressure headache of moderate severity with no obvious triggers. Accompanying the headache was recurring sporadic episodes of right eye amaurosis fugax described as a curtain of vision descending and covering up the visual field of the right eye for 60 seconds, on average two to three times a week. At different times he would also develop transient visual obscurations (TVO) of his left eye lasting up to 10 seconds; these too were sporadic with similar frequency, and described as cloudiness of vision. No constitutional symptoms, such as fever, chills, malaise, myalgias or weight loss, and no other neurologic symptoms such as imbalance, diplopia or numbness were reported. He denied a prior history of migraines or tobacco or alcohol use. His past medical history was significant for hypertension, treated with lisinopril 20 milligrams (mg) daily and hyperlipidemia treated with atorvastatin 40 mg daily.

On examination his height was 5 feet 10 inches, weight 200 pounds with a body mass index of 28.3 kg/m^2^. The blood pressure was 139/90 mmHg with a regular pulse of 78 beats per minute. Bilateral carotid artery auscultation was absent for an arterial bruit and cardiac auscultation revealed no murmurs. His balance was broad-based and a cane was used. Tandem-gait was unsteady and Romberg sign was absent. Heel and toe-standing was achievable. Cranial nerve examination revealed full ocular motion without nystagmus. Visual field testing was full to confrontation, monocular and binocular. Pupils were reactive, symmetric with a normal consensual light reflex and normal accommodative reflex. Visual acuity was 20/20 in both eyes. Pupil dilation was performed by an ophthalmologist and the optic discs and retina were normal bilaterally. The rest of the cranial nerves were entirely normal. Strength in the arms and legs was normal and graded at 5/5 with the Medical Research Council scale. Standing up from the seated position with the arms folded was normal. Coordination testing with bilateral finger-to-nose and heel-to-shin testing was normal. Deep tendon reflexes were lively except for absent bilateral ankle jerks. Sensory examination in the toes was normal to touch-pressure, joint position sense and pin-prick but reduced to vibratory sense. 

**Figure 1:**
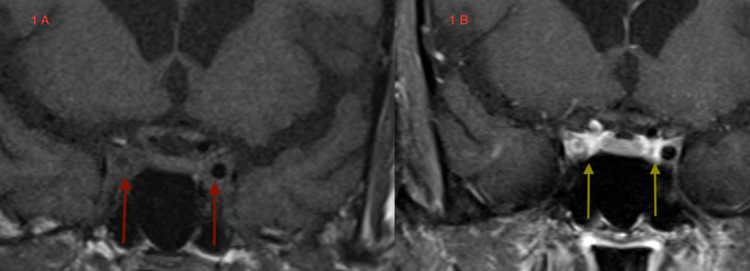
Coronal T1-weighted MRI images. 1A - unenhanced images show carotid siphon arteries in cavernous sinuses (red arrows). 1B - gadolinium enhancement shows intensely enhancing cavernous sinus medial walls (yellow arrows) with luminal narrowing of right carotid siphon. MRI: Magnetic Resonance Imaging

A carotid duplex scan revealed normal peak systolic velocities with no significant stenosis. A magnetic resonance imaging (MRI) of the brain and orbits with and without gadolinium revealed enhancing cavernous sinuses bilaterally with spread of enhancement into the right orbital apex. The right carotid siphon lumen was narrowed with non-occluded superior ophthalmic veins bilaterally (Figure 1).

Sedimentation rate, C-reactive protein (CRP) and a complete blood count were normal. Cerebrospinal fluid (CSF) testing revealed an elevated protein of 81.3 mg per deciliter (normal < 44) and no pleocytosis. Oligoclonal bands and intrathecal synthesis of immunoglobulins and CSF cytology for malignant cells were absent. Based upon the enhancing cavernous sinuses, the mildly inflammatory CSF and the symptomatology of headaches and visual disturbances, a putative diagnosis of probable THS was made. A trial of prednisone at 20 mg twice daily was initiated and by the fourth day the headache had completely resolved. By the end of the second week, the spells of amaurosis fugax of the right eye and transient visual obscurations of the left eye had also completely resolved. The patient is on a slow tapering dose of prednisone with a goal of a maintenance dose of 10 mg daily. The exquisite response to prednisone is also concordant with the diagnosis of THS.

However, several questions are raised, for instance, how do we explain the spells of TVO and amaurosis fugax? The narrowing of the right carotid siphon lumen may indicate a periarteritis of the carotid artery and this may explain the amaurosis fugax, which we discuss in detail in the Discussion section. The transient visual obscurations may be due to inflammation of the orbital apex and inflammation of the optic nerve sheath. This is a speculative but tentative explanation. 

## Discussion

Orbital pseudotumor (OPT), also known as orbital myositis, is a space-occupying inflammatory disease of the orbits that overlaps with THS. It has a similar presentation with several notable exceptions: involvement of the optic nerve with a drop in visual acuity, direct involvement of the ocular muscles and far easier amenability to tissue diagnosis. Twenty-five percent of cases are bilateral and 80% of cases respond to steroid therapy. Radiation therapy and other immuno-suppressive agents are also beneficial [10]. Pathologically, three types are recognised: a lymphoid variant that is steroid-responsive, a granulomatous-variant that is radiation-sensitive and a sclerosing variant that is neither steroid- or radiation-sensitive. The lymphoid and granulomatous variants can pathologically transform to the sclerosing variant [11].

In a comparison of six cases of THS and seven cases of orbital myositis, only the latter group demonstrated hypertrophy of extraocular eye muscles on thin-slice computerized axial tomography of the orbits. The THS group was younger and more responsive to steroids [12]. OPT is also a diagnosis of exclusion; both Graves' disease (thyroid ophthalmopathy) and lymphoproliferative disorders need to be ruled out. Furthermore, cavernous sinus extension has been described [13].

In a study of 126 patients with cavernous sinus syndrome from Spain, tumors (pituitary adenomas, meningiomas) and vascular causes (carotid siphon aneurysm, carotid-cavernous fistula) were the most frequent etiologies, followed by THS which occurred at a rate of 13%. In the THS group, there was a preponderance of females in 62% of cases with a mean age of onset of 56.7 years. Orbital pain was seen in 81% of patients with THS and 50% experienced diplopia. Of these, 64% presented with a third cranial nerve palsy, 28% a fourth cranial nerve palsy and 76% a sixth nerve palsy. Only 4% complained of facial paresthesias over the trigeminal ophthalmic distribution. No optic nerve involvement occurred in the THS group and an MRI abnormality was noted in 95% of cases. Pain at the onset of disease has an odds ratio (OR) of 12.09 (95% confidence interval [CI] 3.14-46.50) and developing third cranial nerve palsy an OR of 4.9 (95% CI 1.01-24.60), both independent associations with THS [14].

In a study of 73 patients with cavernous sinus syndrome from India, tumors, fungal infections and THS were the most common etiologies. Fungal infections were correlated with diabetes and bony erosion. Orbital apex involvement was more correlated with THS than a tumorous etiology [15]. In a study of 151 patients with cavernous sinus syndrome, the most common cause was tumorous and trauma was the most common cause when surgical intervention was included in the analysis. THS was the third most frequent etiology. Infectious etiologies, carotid siphon aneurysm and carotid-cavernous fistula came in fourth [16]. 

In both THS and OPT, there is no bone destruction or hyperostosis, features more typical of invasive malignancies and destructive infectious etiologies. The superior ophthalmic vein is spared in THS and OPT but is otherwise involved in cavernous sinus thrombosis and carotid-cavernous fistula. The lateral wall of the cavernous sinus is slightly convex; the convexity is transformed into a concavity with expanding lesions such as aneurysms or tumors. Useful radiologic signs include carotid siphon narrowing (periarteritis), occlusion of the superior ophthalmic vein (thrombosis, fistula), opacity of the cavernous sinuses and erosion of the sella [17]. In a study of 15 patients with 20 attacks using MRI imaging, cavenous sinus wall enhancement was seen in most attacks, 15 initial and five recurrent. Spread to the orbital apex was seen in 13 attacks and to the superior orbital fissure in seven. Concavity of the lateral sinus wall with enlargement of the cavernous sinus paralleled cavernous sinus enhancement and carotid siphon narrowing was seen in seven attacks [18]. 

Of historical interest, in a study of 26 patients with THS, 16 patients had normal orbital venography. Orbital venography was a technique that involves injecting contrast media into the angular or frontal vein of the face. The anterior facial veins and jugular veins are then compressed in order to allow the dye to flow into the superior opthalmic veins and into the cavernous sinuses. If the facial or frontal vein cannot be accessed, then an internal jugular approach into the inferior petrosal sinus and thence into the cavernous sinus and orbital veins. This technique was particularly useful prior to the MRI era as orbital venous drainage was less variable and anomalous than the arterial supply [19].

## Conclusions

As we outline in the Discussion section, the IHS criteria for THS are narrow and restrictive and they only offer a template for a simple diagnostic algorithm. However, nature does not follow templates and forme frustes (variations) are frequent. Our case highlights the shortcomings of rigid criteria. For instance, carotid siphon arteritis is well documented in THS and therefore it stands to reason that embolic events of an inflamed artery in the territory of the ophthalmic artery may lead to amaurosis fugax or to other visual manifestations. We suggest that a neuroanatomical provision, such as carotid siphon involvement, be added to the IHS criteria for the THS. This may also apply to other headache syndromes as breathtaking advances in neuroradiology, including diffusion tensor imaging and the connectome maps of the brain, unfold in real time. Finally, our case highlights the beauty and power of neuroanatomy as THS provides a quintessential correlation between neuroanatomy, function and localization of disease.
